# Transient Psychotic Symptoms Induced by Acute Sleep Deprivation in a Factory Worker: A Case Report

**DOI:** 10.7759/cureus.95575

**Published:** 2025-10-28

**Authors:** Albert Gassull

**Affiliations:** 1 Emergency Medicine, Hospital Sant Joan de Reus, Tarragona, ESP

**Keywords:** acute psychosis, case report, circadian rhythm, factory worker, mental health, occupational health, shift work, sleep deprivation, transient psychosis, work-related stress

## Abstract

Shift work sleep disorder and acute sleep deprivation are increasingly recognized occupational hazards, particularly among factory workers on rotating schedules, with potential for severe neuropsychiatric manifestations, including transient psychosis. We report the case of a 37-year-old male factory worker with no psychiatric history who developed vivid auditory hallucinations and intense anxiety following consecutive night shifts and daytime overtime, resulting in profound sleep restriction. Presentation to occupational health revealed an exhausted, oriented patient with mild psychomotor agitation but no neurological deficits or substance intoxication. Comprehensive evaluation excluded organic causes through normal laboratory tests and clinical examination. Management consisted of enforced rest and temporary removal from night duties, leading to complete symptom resolution within three days without pharmacotherapy. No recurrence occurred over a three-month follow-up. This case underscores the vulnerability of shift workers to sleep deprivation-induced psychosis, a reversible condition often overlooked in occupational settings. Early recognition and non-pharmacologic intervention can prevent escalation, highlighting the need for sleep hygiene protocols and shift scheduling reforms in industrial environments to mitigate such risks.

## Introduction

Shift work, prevalent in manufacturing sectors, disrupts circadian rhythms and sleep architecture, affecting up to 20% of the global workforce and contributing to shift work sleep disorder [1]. Acute sleep deprivation from irregular schedules can precipitate cognitive impairment, mood disturbances, and, rarely, psychotic symptoms mimicking schizophrenia or delirium [2]. Factory workers face heightened risk due to mandatory overtime and inflexible rotations, with night shifts particularly disruptive to melatonin regulation and rapid eye movement (REM) sleep.

Psychotic features from sleep loss, such as auditory hallucinations, paranoia, and anxiety, are typically transient, resolving with restoration of normal sleep cycles, distinguishing them from primary psychiatric disorders [3]. Mechanisms involve prefrontal cortex hypoactivity, dopamine dysregulation, and stress hormone surges, akin to findings in total sleep deprivation experiments [4]. Despite awareness in sleep medicine, occupational health often underdiagnoses these presentations, attributing them to fatigue rather than intervening promptly.

In Spain and similar systems, workers’ compensation evaluations prioritize physical injuries, potentially delaying recognition of neuropsychiatric effects [5]. This case illustrates isolated psychosis from acute sleep restriction in a healthy individual, emphasizing occupational medicine’s role in prevention. Reporting such events raises awareness for policy changes, as untreated cases may lead to chronic insomnia or misdiagnosis. Early de-escalation without antipsychotics preserves function and avoids iatrogenic risks.

## Case presentation

A 37-year-old male factory worker presented to our occupational health clinic reporting two nights of distressing, vivid auditory hallucinations and severe anxiety. Employed in furniture assembly for 12 years, he had recently completed four consecutive night shifts (22:00-06:00) followed by one day of voluntary daytime overtime (08:00-18:00). He described hearing accusatory voices commenting on his work performance and feeling paranoid about colleagues’ intentions, with episodes peaking during attempted daytime rest. No prior psychiatric history, substance use (denied alcohol, cannabis, or stimulants), head trauma, or family mental illness was reported. The patient also denied any burnout symptoms [6] or workplace mobbing [7]; the overtime was initially voluntary but became non-cancelable after commitment, primarily due to scheduling constraints rather than coercive practices. Medications included none; he occasionally used over-the-counter caffeine tablets for alertness.

On examination, the patient appeared exhausted with dark circles under the eyes and mild psychomotor agitation (fidgeting, rapid speech), but was fully oriented to person, place, time, and situation. Vital signs were stable. Neurological examination showed no focal deficits, tremors, or ataxia; pupils were equal and reactive without nystagmus. Mental status revealed anxious affect but intact cognition (Mini-Mental State Examination (MMSE) score of 28/30), insight into symptoms as “unreal,” and no suicidal ideation. Mood was euthymic at baseline with superimposed acute anxiety. No signs of intoxication or withdrawal were evident, as shown in Table 1.

**Table 1:** Timeline of sleep patterns and symptom development.

Date/Event	Sleep duration (hours/24 hours)	Work schedule	Symptoms reported	Intervention
Days 1–4 (night shifts)	3–4 hours	22:00–06:00 (four consecutive nights)	Fatigue, irritability	None
Days 5–6 (overtime)	3–4 hours	08:00–18:00 (daytime overtime)	Initial anxiety, mild auditory whispers	None
Day 7 (presentation)	~2 hours	Off-duty (attempted rest)	Vivid accusatory auditory hallucinations, intense paranoia	Occupational health evaluation
Days 8–10 (rest period)	8–10 hours/night	Work suspension	Progressive symptom resolution	Enforced rest, no night shifts
Week 2+ (follow-up)	7–8 hours/night	Day shifts only (no overtime)	Complete resolution, normal mood	Shift accommodations

The patient’s reported sleep restriction over approximately 72 hours was cumulative partial deprivation rather than total wakefulness, which is survivable as the body involuntarily enters microsleep episodes, brief, uncontrollable lapses into sleep that mitigate some effects but may go unnoticed by the individual, allowing persistence of severe symptoms such as hallucinations despite preventing immediate lethality.

Diagnostic assessment

Diagnostic methods included a comprehensive clinical examination, mental status evaluation (MMSE score of 28/30), vital signs monitoring, and laboratory investigations (complete blood count, electrolytes, glucose, liver function tests, and thyroid function tests; see Table 2). A urine toxicology screen was considered but not performed due to patient refusal, with no clinical signs of substance use noted; the patient’s denial of substance use and absence of intoxication signs further supported exclusion of drug-induced states. Additionally, a routine urine toxicology screen performed four months prior by the factory’s occupational prevention service was negative, adding credibility to the absence of substance involvement. No additional symptoms suggestive of other etiologies (e.g., fever, seizures, cognitive decline beyond acute fatigue) were present.

**Table 2:** Comprehensive laboratory findings.

Parameter	Value	Units	Reference range
Hematology (complete blood count)			
Hemoglobin	15.1	g/dL	13.5–17.5
Hematocrit	45.2	%	40.0–50.0
Red blood cell count	5.1	× 10¹²/L	4.5–5.9
White blood cell count	6.2	× 10⁹/L	4.5–11.0
Platelet count	280	× 10⁹/L	150–450
Electrolytes			
Sodium	139	mmol/L	135–145
Potassium	4.2	mmol/L	3.5–5.0
Chloride	102	mmol/L	98–106
Bicarbonate	25	mmol/L	22–28
Renal function tests			
Blood urea nitrogen	14	mg/dL	7–20
Creatinine	0.9	mg/dL	0.7–1.3
Glucose			
Glucose	92	mg/dL	70–110 (fasting)
Liver function tests			
Alanine aminotransferase	28	U/L	7–56
Aspartate aminotransferase	22	U/L	10–40
Thyroid function tests			
Thyroid-stimulating hormone	1.8	mIU/L	0.5–4.0

Diagnostic challenges involved differentiating the presentation from delirium (e.g., due to infection or metabolic imbalance), substance-induced psychosis, or primary psychiatric disorders such as schizophrenia (Diagnostic and Statistical Manual of Mental Disorders, Fifth Edition (DSM-5) code: F20.9; International Classification of Diseases, Eleventh Revision (ICD-11) code: 6A20). These were addressed through the normal laboratory results (Table 2), absence of neurological deficits or intoxication signs, lack of psychiatric history, and the temporal association with sleep deprivation. The patient’s preserved insight did not indicate pseudo-hallucinations, as true hallucinations can occur with insight in acute, reversible states such as sleep deprivation-induced psychosis, distinguishing them from chronic conditions where insight is often impaired. While some definitions classify experiences with insight as pseudo-hallucinations, in this occupational context, the vivid, distressing nature aligns with true hallucinations reported in sleep deprivation literature.

The diagnosis was transient psychotic symptoms induced by acute sleep deprivation (consistent with DSM-5 brief psychotic disorder, F23; ICD-11 acute and transient psychotic disorder, 6A23), with other differentials excluded based on the clinical evaluation and rapid symptom resolution following rest, as shown in Table 3. Prognostic characteristics included a favorable outlook given the reversible nature of the condition and absence of comorbidities.

**Table 3:** Summary of differential diagnoses and rationale for exclusion. DSM-5 = Diagnostic and Statistical Manual of Mental Disorders, Fifth Edition; ICD-11 = International Classification of Diseases, Eleventh Revision; EEG = electroencephalography; MMSE= Mini-Mental State Examination; CBC = complete blood count; HIV = human immunodeficiency virus; VDRL = Venereal Disease Research Laboratory; WBC = white blood cells

Differential diagnosis	Key features considered	Reason not pursued/excluded
Delirium (e.g., due to infection, metabolic imbalance)	Fluctuating consciousness, disorientation, fever	Normal labs (electrolytes, glucose), no fever or disorientation; rapid resolution with rest
Substance-induced psychosis (e.g., amphetamines, cocaine, cannabis)	History of use, intoxication signs	Patient denial, no clinical signs; prior negative urine toxicology four months ago; no additional symptoms such as tremors or withdrawal
Schizophrenia or chronic psychosis (DSM-5: F20.9; ICD-11: 6A20)	Persistent symptoms, negative features, chronicity	No prior history, symptoms resolved in three days; three-month follow-up was negative
Temporal lobe epilepsy	Seizures, focal neurological deficits	No seizures or focal deficits; EEG not indicated due to no suggestive features and logistical barriers (hospital referral required)
Encephalitis	Fever, meningism, altered consciousness	No fever or meningism; lumbar puncture not pursued due to absence of symptoms and system constraints
Dementia or incipient dementia	Progressive cognitive decline, memory loss	Age 37 years, MMSE 28/30; rapid resolution; full neuropsychological assessment not indicated/logistically feasible in an occupational setting
Intellectual disability (mental disability)	Lifelong cognitive impairment	No history, intact baseline cognition; not suggested by presentation
Personality disorder	Enduring patterns of behavior	No prior psychiatric history; acute onset and resolution were inconsistent
Vitamin deficiencies (e.g., B vitamins)	Neurological symptoms such as neuropathy	Normal labs (CBC, etc.); no additional features; extended bloodwork not indicated
Hormonal changes (e.g., testosterone, prolactin, hypocretin)	Endocrine symptoms, sleep disorders	No suggestive signs; thyroid normal; not routine in this context
Infectious/autoimmune (e.g., HIV, VDRL, hepatitis, tuberculosis, autoimmune diseases)	Systemic symptoms, serologies	No fever, rash, or risk factors; normal WBC/liver function; serologies not indicated due to lack of symptoms
Other organic (e.g., brain mass, ceruloplasmin/iron studies)	Focal deficits, imaging abnormalities	No neurological signs; brain MRI not pursued due to resolution and access issues

Given the patient’s age (37 years) and near-normal MMSE (28/30), evaluations for incipient dementia, intellectual disability, or personality disorders were not indicated, as symptoms resolved rapidly without suggestive features. Similarly, advanced neuroimaging (e.g., brain MRI), electroencephalography (EEG), or lumbar puncture were not pursued due to logistical constraints in Spain’s occupational health system, such tests are typically hospital-based and reserved for emergency referrals or persistent symptoms, and the clinical picture was not suggestive of temporal lobe epilepsy (no seizures), encephalitis (no fever, meningism), or other organic causes requiring them.

Therapeutic intervention

Initial management advised immediate rest, suspension from night shifts for two weeks, and referral to primary care for follow-up. Laboratory investigations included complete blood count (normal: hemoglobin of 15.1 g/dL, white blood cell count of 6.2 × 10^9^/L), electrolytes, glucose (92 mg/dL), thyroid function (thyroid-stimulating hormone of 1.8 mIU/L), as shown in Table 2. Urine toxicology screen was not performed due to the unwillingness of the patient, but no indirect signs of drug use exposure were noted. EEG was considered if a relapse occurred.

Follow-up and outcomes

Clinician-assessed outcomes included complete resolution of psychotic symptoms (auditory hallucinations and paranoia) and anxiety within three days of enforced rest, with normalized mood reported by the patient. At one-month and three-month follow-ups, no relapse occurred, and sleep logs confirmed consistent seven to eight hours of nightly sleep. Important follow-up diagnostic tests were not required due to full recovery, though an EEG was considered if symptoms recurred (none did). Intervention adherence (to enforced rest and shift accommodations) was assessed through daily occupational health visits during the initial two weeks post-presentation and patient self-reports during those visits, confirming compliance with no difficulties noted. Tolerability was high, as the patient resumed daytime shifts without issues. No adverse or unanticipated events were noted during follow-up.

## Discussion

This case demonstrates transient psychosis triggered by acute sleep deprivation in a shift-working factory worker, resolving spontaneously with rest alone, a pattern consistent with sleep loss-induced neuropsychiatric decompensation [1]. Unlike chronic schizophrenia, these symptoms lack negative features or chronicity, instead reflecting reversible neurochemical imbalances from prolonged wakefulness [2]. Auditory hallucinations, as seen here, arise from REM sleep pressure accumulation, with prefrontal-amygdala disconnect impairing reality testing [3].

Factory environments exacerbate risks through inflexible scheduling and peer pressure for overtime, leading to cumulative sleep debt [4]. Studies show night shift workers experience 20-30% reduced sleep efficiency, with vulnerability to psychosis after 48-72 hours of restriction, mirroring experimental total sleep deprivation paradigms [8]. Our patient’s four-night shift sequence plus overtime aligns with this threshold, without confounding substances or comorbidities.

Diagnostic challenges include differentiation from delirium, drug-induced states, or primary psychosis. In this case, normal examinations, toxicology, and rapid resolution supported functional etiology [9]. While extended bloodwork (e.g., vitamins, hormones, serologies) or advanced testing (e.g., EEG, lumbar puncture, neuroimaging) could theoretically rule out rare differentials such as vitamin deficiencies, autoimmune diseases, temporal lobe epilepsy, or encephalitis, these were not indicated given the absence of suggestive features (e.g., no fever, seizures, cognitive decline) and rapid resolution with rest alone. In Spain’s public health system, such tests are not routinely accessible in occupational settings without hospital referral, and the three-month follow-up showed no progression to chronic psychosis (e.g., schizophrenia), consistent with literature indicating low risk in isolated sleep deprivation episodes among healthy individuals. Occupational health’s frontline role is crucial, as primary care may overlook work-related triggers. Non-pharmacologic intervention succeeded here, avoiding the side effects of antipsychotics such as sedation or extrapyramidal symptoms, which could impair return to work [10].

Literature gaps persist: while military and laboratory studies document psychosis from extreme deprivation, real-world occupational cases are underreported, particularly in manufacturing [11]. Prevalence may be noteworthy among shift workers during peak deprivation, yet screening tools such as the Epworth Sleepiness Scale are rarely routine [12]. This report’s novelty lies in isolated auditory phenomena without delirium, in a non-substance-using individual, highlighting pure sleep-mediated pathways possibly involving elevated cortisol and dopamine hypersensitivity [13].

Limitations include subjective sleep reporting and lack of polysomnography or baseline EEG, though clinical response was diagnostic. Future research should validate biomarkers (e.g., salivary melatonin) for early detection. Occupational interventions, such as shift limits, naps, and education, could prevent such episodes, reducing absenteeism and errors. Clinicians must advocate for sleep as an occupational hazard, integrating neurology and psychiatry in evaluations. This underscores systemic needs for regulated rest in high-risk industries.

Patient perspective

The patient expressed relief following the period of enforced rest, noting that his symptoms felt “unreal” during the episode but fully resolved afterward. He appreciated the adjustment to daytime shifts, which allowed him to maintain regular sleep and return to work without ongoing anxiety or performance concerns.

## Conclusions

Acute sleep deprivation from shift work can precipitate reversible psychosis, as evidenced by this factory worker’s full recovery with rest alone. Prompt occupational intervention prevented chronicity and medication use. Enhanced awareness and policy reforms are essential to safeguard shift workers from such underrecognized risks. In summary, recognizing sleep deprivation as a reversible trigger for transient psychosis can guide non-invasive management and prevent misdiagnosis.
